# Integrated miRNA and mRNA Transcriptome Analysis Reveals Regulatory Mechanisms in the Response of Winter *Brassica rapa* to Drought Stress

**DOI:** 10.3390/ijms251810098

**Published:** 2024-09-20

**Authors:** Li Ma, Yanxia Xu, Xiaolei Tao, Abbas Muhammad Fahim, Xianliang Zhang, Chunyang Han, Gang Yang, Wangtian Wang, Yuanyuan Pu, Lijun Liu, Tingting Fan, Junyan Wu, Wancang Sun

**Affiliations:** State Key Laboratory of Aridland Crop Science, College of Agronomy, Gansu Agricultural University, Lanzhou 730070, China; mal@gsau.edu.cn (L.M.); xyx7991@163.com (Y.X.); txl162185@163.com (X.T.); fahimabbaskhan@yahoo.com (A.M.F.); 18219919034@163.com (X.Z.); hannn216@163.com (C.H.); yangang1018@163.com (G.Y.); wangw@gsau.edu.cn (W.W.); vampirepyy@126.com (Y.P.); liulj198910@163.com (L.L.); fantt@gsau.edu.cn (T.F.)

**Keywords:** winter *Brassica rapa*, transcriptome sequencing, miRNA sequencing, drought stress, differential expression

## Abstract

Drought is a major abiotic stress factor that reduces agricultural productivity. Understanding the molecular regulatory network of drought response in winter rape is of great significance for molecular *Brassica rapa*. In order to comprehensively analyze the network expression of DEGs and DEMIs in winter rape under drought stress, in this study we used Longyou 7 as the experimental material to identify DEGs and DEMIs related to drought stress by transcriptome and miRNA sequencing. A total of 14–15 key differential mRNA genes related to drought stress and biological stress were screened out under different treatments in the three groups. and 32 differential miRNAs were identified through targeted regulatory relationships, and the mRNA expression of 20 target genes was negatively regulated by the targeting regulatory relationship. It is mainly enriched in starch and sucrose metabolism, carbon metabolism and other pathways. Among them, gra-MIR8731-p3_2ss13GA18GA regulated the expression of multiple mRNAs in the three treatments. miRNA is mainly involved in the drought resistance of Chinese cabbage winter rape by regulating the expression of target genes, such as starch and sucrose metabolism, amino acid biosynthesis, and carbon metabolism. These miRNAs and their target genes play an indispensable role in winter rapeseed drought stress tolerance regulation.

## 1. Introduction

Rapeseed is among one of the most important oil seed crops globally and in China, with a perennial cultivation area of approximately 170,000 hectares only in Gansu province of China. The primary cultivation regions of rapeseed are distributed in the arid and semi-arid regions of central Gansu Province of China. Global warming causes oilseed rape to experience many environmental pressures during its growth cycle. Among these stresses, drought stress has the most significant impact on the plant’s growth, development, physiology, biochemistry, and overall metabolism process [1,2]. Drought poses a significant threat to agriculture, severely impeding the growth, development, and overall productivity of crops on a global scale. Drought stress occurs when there is an imbalance between the water demand and supply of plants, leading to inadequate water in the plant body and causing harm to the plant [3,4]. Under drought stress, plants enhance their osmoregulation ability by increasing the levels of proline and soluble protein. Additionally, plants produce various antioxidant enzymes, including superoxide dismutase, peroxidase, and catalase, to eliminate excessive reactive oxygen species and mitigate the impact of drought stress [5,6,7]. Hence, comprehending the molecular regulatory network of drought response holds immense importance for molecular breeding. Research has demonstrated that plants respond to drought stress through intricate mechanisms that occur at both the transcriptional and post-transcriptional levels. The response entails a sequence of intricate molecular processes, including abscisic acid (ABA)-related signaling, elevated levels of endogenous ABA during drought stress, and the activation of ABA-dependent and ABA-independent transcriptional regulatory networks [8,9]. Furthermore, a class of naturally occurring non-coding RNAs known as miRNAs play a vital role in regulating gene expression at the post-transcriptional level during abiotic stress in plants [10].

The micro-RNA (miRNA) is an endogenous non-coding RNA containing 20–25 nucleotides and is ubiquitous in plants [11]. Prior studies have demonstrated that miRNAs mainly play a role at the post-transcriptional level and are involved in the regulation of plant growth and development, stress response, and hormone signal transduction by negatively regulating the expression of plant genes [12]. miRNAs interact with target cell mRNAs by forming base complementary pairs to control gene expression. Advancements in sequencing technology have recently resulted in the identification of novel drought-responsive genes and small RNAs in plants. Transcriptome sequencing, also known as mRNA-Seq, has effectively been used to examine gene expression patterns in different plants experiencing drought stress. Transcriptome studies conducted on *Arabidopsis thaliana* have revealed that various genetic backgrounds exhibit significant responses to mild drought stress during leaf development. The response to drought stress involves abscisic acid signaling, proline metabolism, and cell wall modification [13]. Research on potatoes has demonstrated that the regulation of metabolic pathways DEGs can lead to responses to drought and rewatering. These DEGs are responsible for the production of endogenous hormones and the transmission of signals in the plant [14]. A thorough examination of gene expression profiling revealed that specific genes triggered by drought in rice, as well as genes with greater expression levels in the drought-tolerant genotype under both normal and drought stress conditions, collectively contributed to the enhancement of drought-stress tolerance [15].

To understand the effects of drought stress on major oilseed crops such as soybean, genome-wide transcriptome analysis of leaf tissues from two contrasting soybean lines, drought-susceptible (DS) and drought-tolerant (DT), and pairwise comparisons of DS and DT lines under drought and control conditions revealed valuable SNP variants in the water channel protein gene of the DT line, which should be useful for improving marker-assisted selection for drought-tolerant soybean [16]. The maize drought transcriptome was investigated using RNA-Seq analysis comparing fertilized ovary and basal leaf meristem tissues subjected to drought treatment and normal watering, and more drought-responsive genes were found in the ovary [17]. The transcriptome differences in the DT infiltration line H471, the DT donor P28 and the drought-sensitive, recurrent parent HHZ under drought stress were investigated by deep transcriptome sequencing, which revealed the molecular genetic pathways of drought stress tolerance as well as co-localization of DEGs with DT-associated QTLs and infiltration compartments and will provide a useful resource for further dissecting the molecular mechanisms of drought stress response in rice [15]. The miRNA expression of two different wheat genotypes was investigated by deep sequencing, and the expression levels of 10 target genes were detected by qPCR analysis and found to be negatively correlated with the levels of the corresponding miRNAs. The results suggest that the differential expression patterns of miRNAs between these two genotypes may play an important role in dehydration stress tolerance in wheat and may be a key factor in determining the level of stress tolerance in different genotypes of wheat [18]. To further understand the role of sRNAs in water deficit response. Sequencing analysis was performed to identify sRNAs regulated in leaves and roots of sugarcane cultivars with different drought sensitivities, and 28 and 36 conserved miRNA families were identified in leaf and root libraries, respectively. Some genes of the sRNA biogenesis pathway were down-regulated in tolerant genotypes and up-regulated in sensitive genotypes in response to water deficit. This study contributes to further understanding of the role of sRNAs in water deficit in sugarcane [19]. In a study, a small RNA library was constructed from polyethylene glycol (PEG 6000)-treated and control potato samples, and a large number of known and novel miRNAs were identified. Four miRNAs were identified as drought-associated regulatory genes by target prediction, annotation, and expression analysis of miRNAs and their putative target genes, and the relative expression trends in these miRNAs were the same as those predicted by Solexa sequencing and negatively correlated with target gene expression. predicted by Solexa sequencing and were negatively correlated with the expression of target genes. The results provide molecular evidence that miRNAs may be involved in drought response in potato plants [20]. For instance, miRNA 398 is believed to have a direct association with plant stress regulation networks and has the ability to control plant responses to drought and salt stress [21].

Transcriptome sequencing has effectively been used to analyze gene expression patterns in several plants under drought stress [22,23,24]. To date, there have been limited studies analyzing the mRNA and miRNA profiles in oilseed rape subjected to drought stress and subsequent rewatering treatments. In the present study, we utilized miRNA-mRNA co-analysis in rape to reveal that miRNAs might have a significant impact on rape response to drought stress by controlling the expression of target genes (mRNA). Integrating miRNA-mRNA analysis can enhance the comprehension of how crops adapt to adverse environmental conditions [25,26,27]. It will greatly improve our understanding of the molecular regulatory mechanisms underlying plant stress responses [28]. This holds immense importance in the development of drought-resistant cultivars and mitigating drought stress in winter *B. rapa*.

## 2. Results

### 2.1. Global Analysis of Transcriptomics in Strongly Drought-Tolerant Winter Brassica rapa before and after Drought Stress

We carried out transcriptome sequencing of drought-treated rapeseed leaves, and quality control of the sequencing data obtained a total of 60.35 Gb of clean data; all samples were above 5.84 G, and the proportion of effective reads of each sample was about 96.97%. The Q30 base percentage of the nine samples was greater than 97.77%, indicating that the base recognition accuracy was high. The proportion of GC content ranged from 46.50 to 47, indicating no base separation (Supplementary Table S1). Hisat was used to compare the pretreated Valid Data with the reference genome, and the comparison rate was 81.93~82.47%, indicating that the duplication rate between the sequencing samples and the reference genome was high, which met the needs of subsequent analysis (Supplementary Table S2). The number of fragments aligned in different regions of the specified reference genome (exon, intron and intergenic regions) was statistically analyzed, and the results showed in Figure S1 that the percentage of short fragments aligned to exons in the nine samples was more than 94%, which met the needs of further analysis. A total of 14,263 differentially expressed genes were identified in D/CK, of which 5756 were up-regulated and 8507 DEGs were down-regulated. A total of 10,037 differentially expressed genes were expressed in R/CK, of which 5109 were up-regulated and 4928 DEGs were down-regulated. There were 9435 differentially expressed genes in R/D, 6785 up-regulated genes and 2654 down-regulated DEGs.

FPKM (FPKM indicates the number of fragments per kilobase per million mapped reads of an exon model, and in simple terms, the FPKM value can be interpreted as the amount of gene expression.) was used to measure the abundance of genes expressed in different samples. The results showed that the box plots of FPKM values for each sample had similar upper quartiles, medians and lower quartiles, indicating consistent overall gene expression levels across samples. The FPKM density distribution map shows a roughly normal distribution, with the peaks tending to be the same and the area size of the region is 1, which represents the sum of the probabilities of 1. The overall gene expression level distribution curve was relatively consistent, and there were no abnormally high or low expression genes (Figure S2A,B). In addition, the correlation coefficient between the samples was analyzed (Figure S2C,D), and it was found that the reproducibility between the control group and the treatment group was good, and the data were relatively reliable. Principal component analysis showed that the control group and the treatment group showed a tendency to aggregate with each other, and the reproducibility within the group was acceptable, and there was a certain degree of discrimination between the two. The above results generally show that the experimental design is reliable, the transcriptome data meet the sequencing requirements, and the results can be used for subsequent analysis.

### 2.2. Analysis of Differentially Expressed mRNAs (DEGs) in Winter Brassica rapa under Drought and Rewatering Treatments

**Figure 1:**
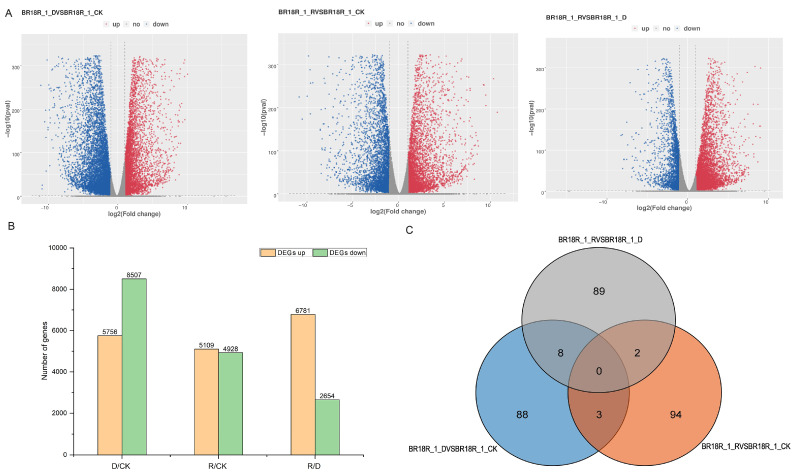
Analysis of differentially expressed mRNAs (DEGs). (**A**), Volcano plot of differential gene expression levels. (**B**), Statistics of up- and down-regulation of differentially expressed genes in different groups. (**C**), Wayne plots of differentially expressed genes.

**Figure 2:**
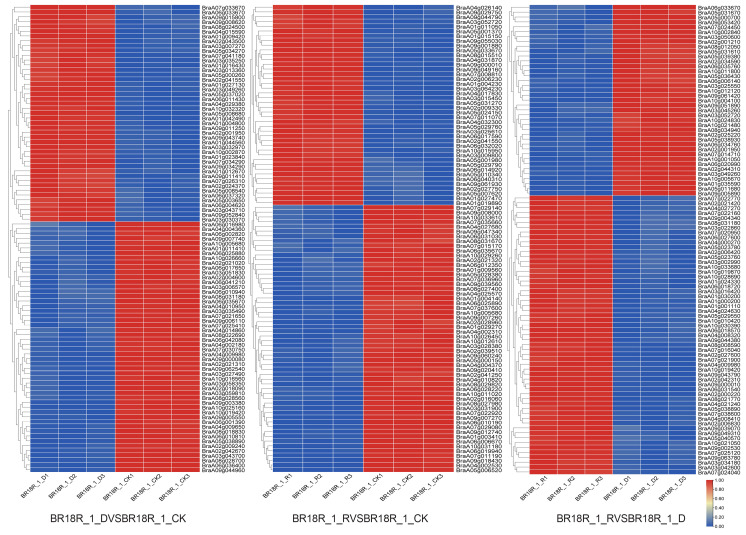
Heatmap of differential expression profiles of all mRNAs in the three treatment groups. Heatmaps were plotted using log2 values for each gene.

In order to comprehensively study the differentially expressed genes in response to drought stress in drought and post-drought rewatering of rapeseed, the *p*-value < 0.05 and log2 fold change > 2 were the standard identification of differentially expressed genes (DEGs), and the results showed that compared with the other two groups, the BR18R_1_D vs. BR18R_1_CK had the most differentially expressed genes, with 14,263 DEGs, of which 5756 DEGs were up-regulated and 8507 DEGs were down-regulated. BR18R_1_D vs. BR18R_1_CK had relatively large numbers of DEGs compared to the other two groups, with 8 DEGs in BR18R_1_D vs. BR18R_1_CK and BR18R_1_R vs. BR18R_1_D; there were 3 overlapping DEGs between BR18R_1_R vs. BR18R_1_CK and BR18R_1_R vs. BR18R_1_CK and 2 overlapping DEGs between BR18R_1_R vs. BR18R_1_D (Figure 1, Supplementary Table S3). The clustering heat map showed that the expression levels of differentially differentiated genes were significantly different after stress (Figure 2).

### 2.3. GO and KEGG Enrichment Analysis of Differential Genes

**Figure 3:**
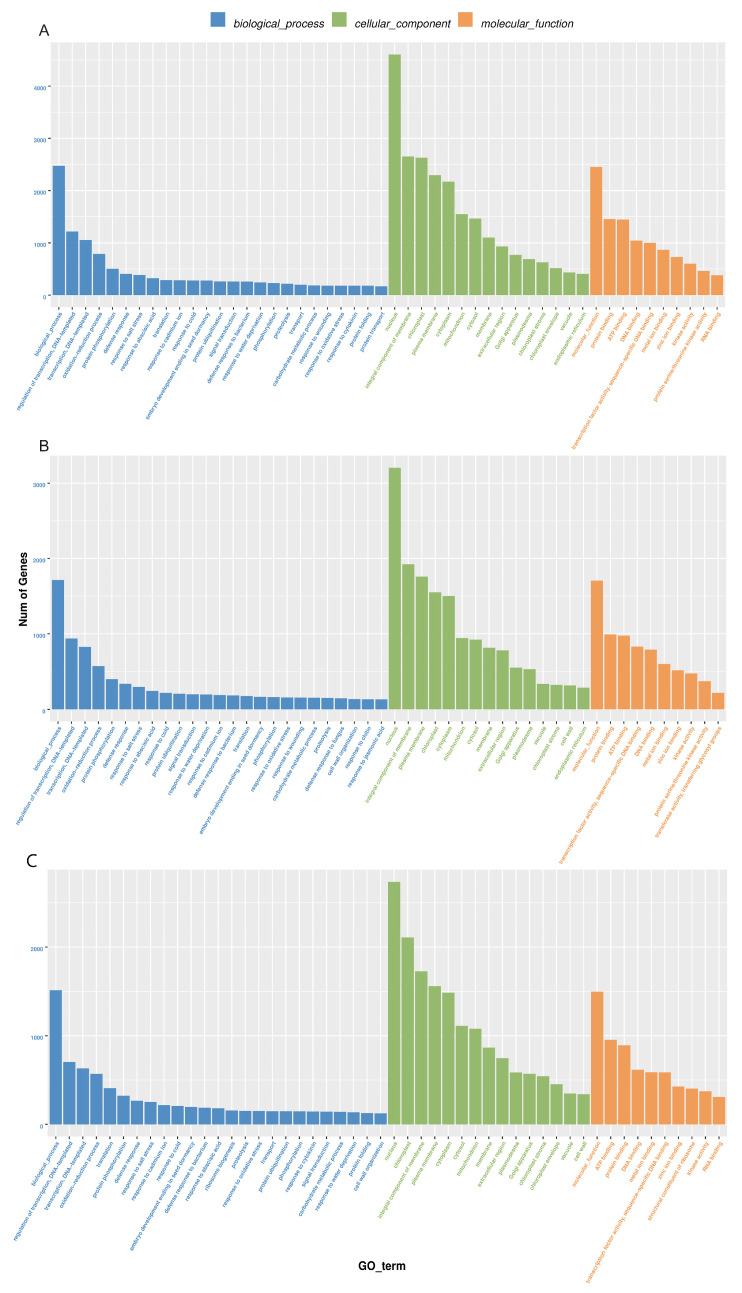
Histogram of GO enrichment of differential genes. (**A**), BR18R_1_D vs. BR18R_1_CK. (**B**), BR18R_1_R vs. BR18R_1_CK. (**C**), BR18R_1_R vs. BR18R_1_D.

GO analysis divides the function of genes into three categories: biological processes, cellular components, and molecular functions. In biological processes, DEGs are mainly involved in DNA-triggered positive and negative regulation of transcription, the oxidation-reduction process, protein phosphorylation, and defense against drought stress and salt stress (Figure 3, Supplementary Table S4). The drought stress defense process includes: “GO:0009737: response to abscisic acid”, “GO:0006952: defense response” and “GO:0050832: defense response to fungi”. The entries enriched in DEGs in cell components are mostly related to the nucleus, chloroplast, cytoplasm, and mitochondria. DEGs are mainly enriched in protein binding, ATP binding, DNA binding, transcription factor activity, metal ion zinc ion binding, kinase activity and other items in molecular function, and drought defense includes: “GO:0016301: kinase activity”. In the GO enrichment histogram BR18R_1_DVSBR18R_1_CK the number of treated genes was the largest.

**Figure 4:**
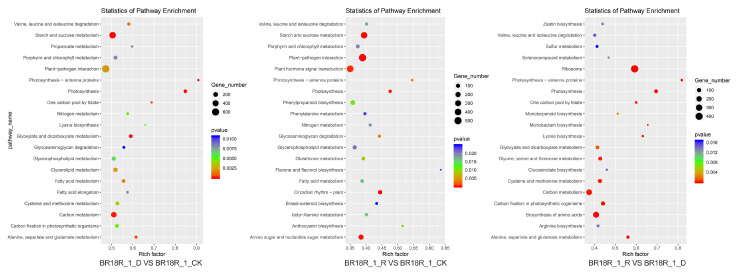
Scatter plot of KEGG enrichment of differential genes.

The main pathways involved by the differential genes were analyzed by KEGG enrichment analysis and found that after BR18R_1_D vs. BR18R_1_CK, DEGS was significantly enriched in “ko00500: starch and sucrose metabolism”, “ko01200: carbon metabolism”, and “ko04626: interaction between plants and pathogens”. After BR18R_1_R vs. BR18R_1_CK treatment, its differential genes DEGs were mainly enriched in “ko00500: starch and sucrose metabolism”, “ko04626: interaction between plant and Pathogen” and “ko04075: phytohormone signaling”. After BR18R_1_R vs. BR18R_1_D treatment, the differential genes DEGs were mainly enriched in “ko03010: ribosome”, “ko01200: carbon metabolism” and “ko01230: biosynthesis of amino acids” (Figure 4, Supplementary Table S5).

### 2.4. Analysis of SNV/Indel in Winter Brassica rapa before and after Drought Treatment

**Figure 5:**
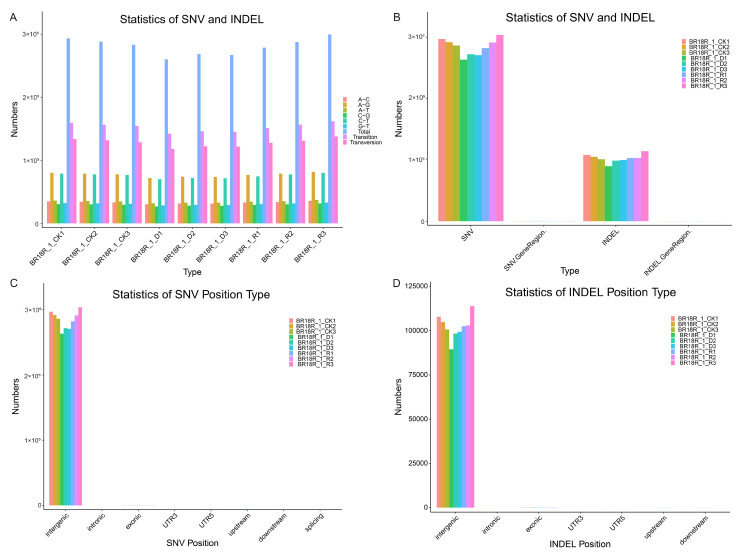
Analysis of SNV/Indel in winter *B. rapa*. (**A**), Statistics of SNV and INDEL variant types. (**B**), Statistics of SNV and INDEL numbers. (**C**,**D**), Classification of SNV and INDEL locations.

To further investigate the key mechanisms of drought resistance response in winter *B. rapa*, we used transcriptome analysis to identify SNVs and INDELs. A total of 2,526,597 SNVs were detected in the transcriptome, of which 1,373,697 were transitions and 1,152,900 were transversions. Among them, the number of SNVs was highest under normal watering without treatment, decreased after drought treatment, and increased again after rehydration treatment (Figure 5A, Supplementary Table S6). The number of SNVs in each sample is greater than the number of INDELs (Figure 5B, Supplementary Table S6). Most SNVs and INDELs are distributed in intergenic regions, with a few in exon regions (Figure 5C,D, Supplementary Table S6). The study of SNVs and INDELs provides valuable resources for further cultivation of drought-resistant varieties of winter *B. rapa*.

### 2.5. Screening and Identification of Key Differential Genes in Drought Stress

**Figure 6:**
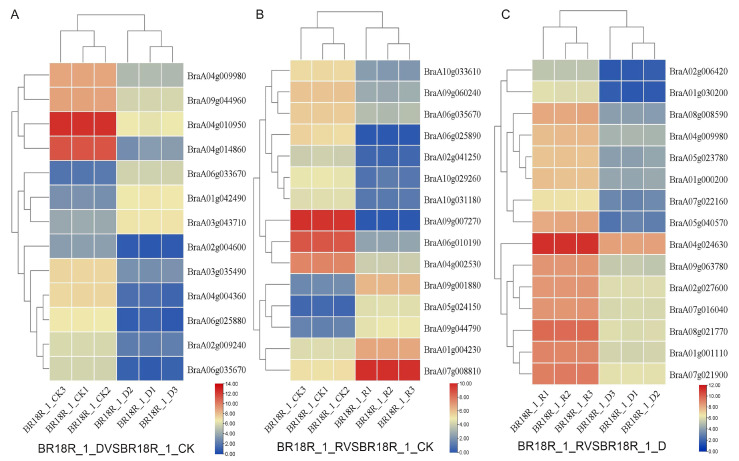
Heatmap of key differential mRNA expression. (**A**), BR18R_1_D vs. BR18R_1_CK. (**B**), BR18R_1_R vs. BR18R_1_CK. (**C**), BR18R_1_R vs. BR18R_1_D. Heatmaps were plotted using log2 values for each gene.

In order to investigate the effect of mRNA on the expression levels of genes related to drought stress and biotic stress in rapeseed, 14–15 differentially expressed mRNAs (DEGs) related to drought stress and various abiotic stresses were selected from the three treatments (Figure 6). The log value of the expression data was calculated, and the expression heat map analysis showed that the expression of BraA01g042490, BraA06g033670 and BraA03g043710 went through upward adjustments of approximately 16, 15 and 7.5 times, respectively, in the BR18R_1_D vs. BR18R_1_CK. BraA04g010950, BraA02g009240, BraA04g004360, BraA04g014860, BraA06g035670, BraA06g025880, BraA03g035490, BraA04g009980, BraA09g044960 gene expression were down-regulated to different degrees; The expression of BraA02g004600 gene was down-regulated from 10.82 to 0.70. BraA06g010190 and BraA04g002530 were down-regulated 88.6 and 12.6-fold in BR18R_1_R vs. BR18R_1_CK treatment, respectively. BraA07g008810, BraA09g001880 and BraA04g002530 genes were up-regulated. In BR18R_1_R vs. BR18R_1_D, the expression levels of BraA05g040570, BraA04g024630, BraA02g006420, BraA01g030200 and BraA07g022160 were significantly up-regulated after drought and rewatering, and the other genes were up-regulated to varying degrees, indicating that rewatering was conducive to crop growth and development after drought treatment.

### 2.6. Analysis of Differentially Expressed miRNAs before and after Drought Treatment

**Figure 7:**
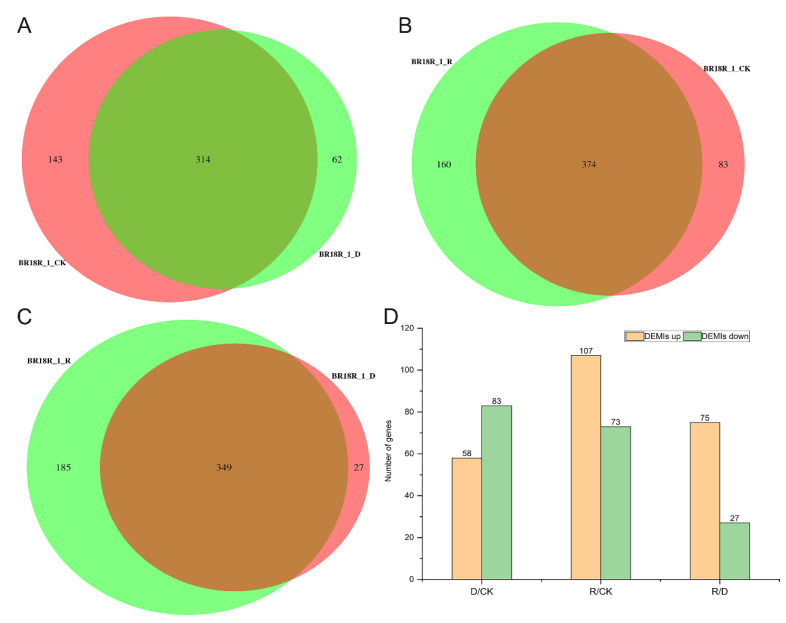
Differentially expressed miRNA analysis and related gene expression statistics. (**A**), Venn diagram of miRNA in BR18R_1_CK and BR18R_1_D. (**B**), Venn diagram of miRNA in BR18R_1_R and BR18R_1_CK. (**C**), BR18R_1_R and BR18R_1_D. (**D**), Differentially expressed genes in different groups.

Additionally, miRNA-seq analysis was used to identify differentially regulated genes under drought stress conditions, and we used DEseq software version 2.12 to compare gene expression between groups of rape under three growing conditions, and found that there were 374 miRNAs between BR18R_1_R and BR18R_1_CK by Venn plot clustering (Figure 7A–C, Supplementary Table S7). There were 314 and 349 miRNAs between BR18R_1_CK and BR18R_1_D, and BR18R_1_R and BR18R_1_D, respectively. A total of 141, 180 and 102 miRNAs were identified in BR18R_1_D vs. BR18R_1_CK, BR18R_1_R vs. BR18R_1_CK and BR18R_1_R vs. BR18R_1_D, respectively. By miRNA sequencing, there were 58 up-regulated differentially expressed miRNAs (DEMIs) and 83 down-regulated differentially expressed miRNAs (DEMIs) in the BR18R_1_D vs. BR18R_1_CK, 107 up-regulated differentially expressed miRNAs (DEMIs) and 73 down-regulated differentially expressed miRNAs (DEMIs) in the BR18R_1_R vs. BR18R_1_CK, and 75 up-regulated differentially expressed genes and 27 down-regulated differentially expressed miRNAs (DEMIs) in the BR18R_1_R vs. BR18R_1_D (Figure 7D). It was shown that the expression of most of the drought-affected genes observed in BR18R_1_R vs. BR18R_1_D samples returned to a “normal” state after rewatering treatment.

### 2.7. GO and KEGG Enrichment Analysis of Differentially Expressed miRNA Target Genes

**Figure 8:**
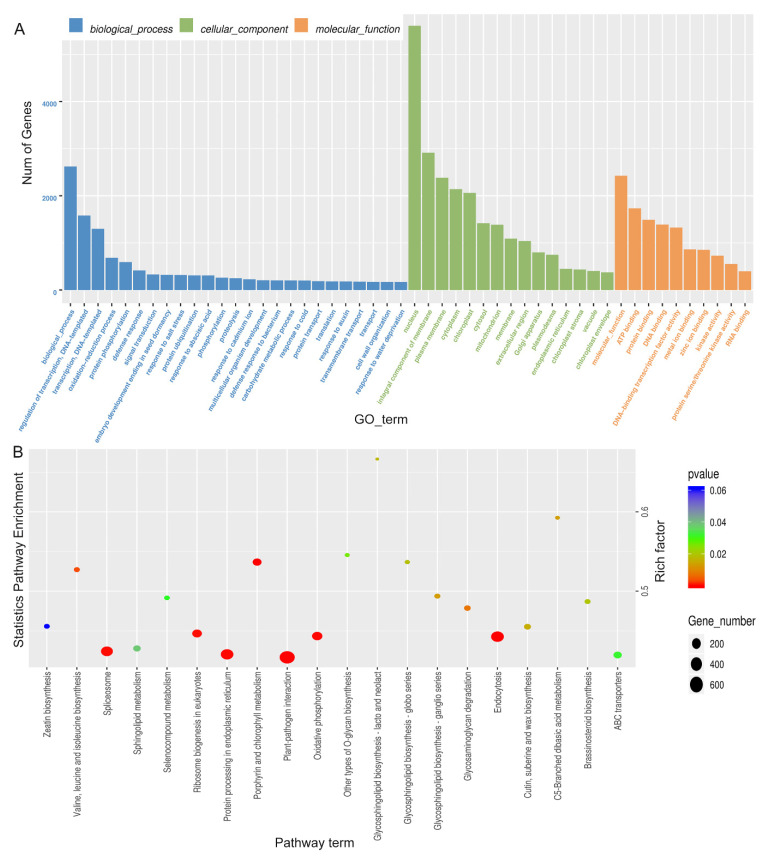
Differential miRNA target gene GO and KEGG enrichment analysis. (**A**), Histogram of GO enrichment. (**B**), Scatter plot of KEGG enrichment properties.

GO analysis of differentially expressed miRNA showed that most of the miRNA target genes were mainly involved in transcriptional regulation, DNA-dependent transcription, redox process and other processes in biological processes. Most of the entries in the enrichment of cellular components are related to the nuclear components and membrane-bound organelles, plasma membrane, and chloroplast. Their molecular functions are mainly enriched to ATP binding, DNA binding, protein binding, etc. (Figure 8A, Supplementary Table S8). The main pathways involved in miRNA target genes were analyzed, and it was found that many target genes were significantly enriched in ribosomal biogenesis, plant pathogen interactions, porphyrin and chlorophyll metabolism, and oxidative phosphorylation (Figure 8B, Supplementary Table S8).

### 2.8. Joint Analysis of DEMIs and DEGs Co-Expression

In order to explore the relationship between miRNA and mRNA under drought stress, the microRNA sequencing data with differential expression changes and mRNA transcriptome data were integrated and analyzed, and the targeting association between microRNA and mRNA was carried out in combination with bioinformatics prediction, and the association regulatory mechanism of DEMIs and DEGs was explored. In the selected DEGs, we found that a total of 8 DEGs were targeted and 10 DEMIs were combined in the BR18R_1_DVSBR18R_1_CK group to form a negative regulatory targeting relationship. BraA04g014860 simultaneously targets gma-miR6300 and gma-miR6300_R+1, and BraA02g004600 simultaneously targets bra-miR9558-3p and gra-MIR8731-p3_2ss13GA18GA. In the BR18R_1_RVSBR18R_1_CK, seven DEGs were targeted and fourteen DEMIs were bound to form a negative regulatory targeting relationship. Among them, BraA02g041250 simultaneously targets and binds eight DEMIs: ath-MIR414-p5_1ss18CT, bra-miR156a-3p, bra-miR156b-3p_1ss15TC, bra-miR172c-3p_R-1, bra-miR172d-3p, gma-miR6300, gma-miR6300_R+1, and gma-miR6300_R+2. There were five DEGs in the BR18R_1_R vs. BR18R_1_D that targeted eight DEMIs. A negative regulatory-targeting relationship was formed. Further analysis of association regulation showed that BraA06g033670 was involved in the regulation and expression in both BR18R_1_D vs. BR18R_1_CK and BR18R_1_R vs. BR18R_1_D. At the same time, BraA06g035670 was expressed in BR18R_1_D vs. BR18R_1_CK and BR18R_1_R vs. BR18R_1_CK (Supplementary Tables S9 and S10).

**Figure 9:**
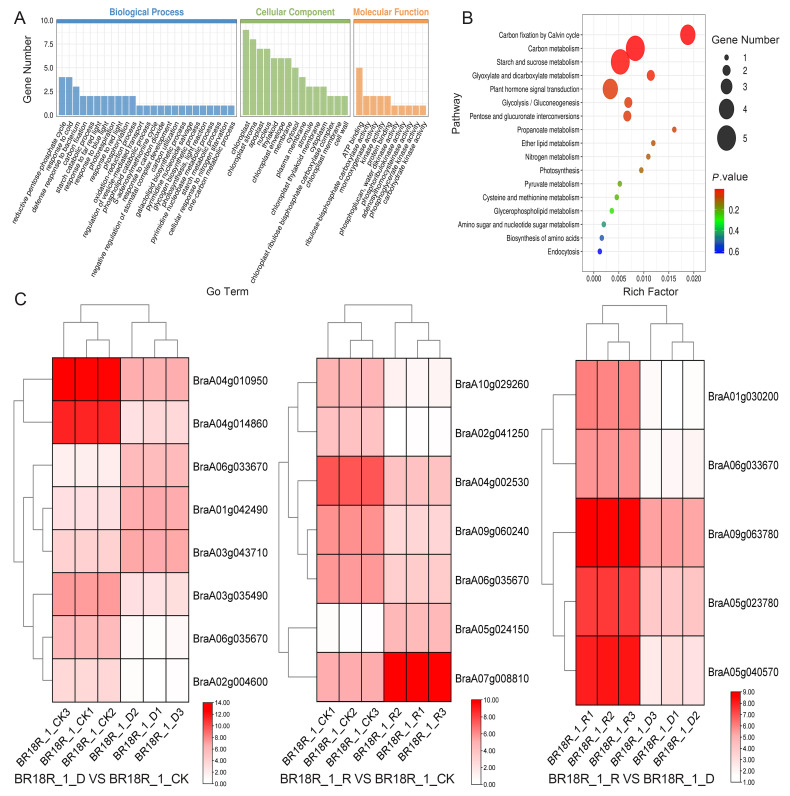
Target mRNA enrichment analysis and expression profiling. (**A**,**B**), GO and KEGG enrichment properties. (**C**), Heatmap of target mRNA expression. Heatmaps were plotted using log2 values for each gene.

Their selected DEGs according to targeting relationships were analyzed for GO and KEGG enrichment and expression. The results showed that most of the target mRNAs were mainly enriched in GO:0019253: reducing pentose phosphate cycle, GO:0009409: response to cold, and GO:0042742: gene defense response to bacteria in biological processes; in cellular components target mRNAs were enriched in chloroplasts, chloroplast stroma, cytoplasm, and nucleus; and the entries enriched in molecular functions mostly related to ATP binding, ribulose-bisphosphate carboxylase activity, monooxygenase activity, kinase activity, and protein binding (Figure 9A,B). By expression heatmap analysis, In BR18R_1_D vs. BR18R_1_CK, the targeted mrna BraA04g0100950 and BraA04g014860 were down-regulated by approximately 66 and 300-fold, respectively, and BraA03g043710 and BraA01g042490 were up-regulated by 7- and 15-fold, respectively. In BR18R_1_R vs. BR18R_1_CK, BRA10g029260 and BraA02g041250 are downgraded by 25- and 37-fold, and both BraA05g024150 and BraA07g008810 are significantly upgraded to 50 and 25 times. In BR18R_1_R vs. BR18R_1_D, the five target mrna of BraA05g023780 and BraA05g040570, BraA09g063780, BraA01g030200, and BraA06g033670 were up-regulated to different degrees, which were, respectively, up-regulated by about 11, 46.8, 9.7, 28.7 and 2.7 times (Figure 9C).

In this experiment, due to the complex regulatory relationship between miRNA and mRNA, one miRNA may have a targeting relationship with multiple mRNAs at the same time, while the expression of one target gene mRNA may be regulated by the post-transcriptional level of multiple miRNAs at the same time. These results suggest that drought is one of the main limiting factors affecting plant growth and development, and plant response to drought stress is a complex signaling regulatory process that is regulated by multiple genes and involves a variety of metabolites and synthetic pathways. The use of transcriptome sequencing technology can quantify gene expression and mine key functional genes, which is an important means to cultivate excellent traits and study gene function.

## 3. Discussion

Abiotic stresses such as drought, high temperature, cold, nutrient deficiency, and high salinity are common in nature, and these stresses have become the key factors restricting plant growth and development and affecting plant yield and quality [4,29,30]. Over the course of long-term evolution, plants have developed a set of active defense mechanisms to resist the effects of adverse environmental influences [31]. Various technologies, such as genomics, transcriptomics, metabolomics, and proteomics, play an important role in exploring the molecular mechanisms of plant growth, development, and response to adversity stress by studying plants at the levels of DNA, transcription, translation, and metabolism [29,32]. Therefore, this study summarized and prospected the response of plants to abiotic stress through transcriptomics and miRNA omics technology, which was a comprehensive reference and laid a theoretical foundation for the cultivation of high-quality and stress-tolerant plant varieties. The miRNA normally silences gene expression by inhibiting translation and accelerating the degradation of the target mRNA [33]. The interaction of miRNA and signaling pathways of growth regulators is not only a regulator of plant growth and development but also involved in regulating the phenotypic plasticity induced by various environmental stimuli such as light, temperature, nutrients and other nutrients, promoting plant evolution and adaptation. Therefore, in this study, the targeting relationship between miRNAs and their target gene mRNAs was identified in three groups of treatments by transcriptomics and miRNAomics, and most of the target mRNA-enriched entries were found to be related to resistance to adversity and response to abiotic stresses by GO and KEGG, which provided a comprehensive reference and theoretical basis for the cultivation of high-quality and adversely tolerant plant varieties.

Transcriptomics was used in soybean (*Glycine max* L.), corn (*Zea mays* L.), apple (*Malus pumila* Mill.), rice (*Oryza sativa* L.) and other plants and has been widely used in research on drought stress [34,35,36,37]. Understanding the mechanism of drought stress tolerance in sweet potato helps to breed good germplasm and select drought-tolerant varieties. Phenotypic and physiological traits of 17 sweet potato breeding lines and 10 varieties were comprehensively analyzed under drought stress by treating them for 48 h in Hoagland medium containing 20% PEG6000. Transcriptome analysis indicated that hormone signaling pathways may play a key role in determining drought tolerance in sweet potato. Wei et al. on maize (*Zea mays* L.) Two drought-tolerant lines and two drought-sensitive lines were treated with drought for 1, 2 and 5 days, and RNA-seq sequencing was performed on maize leaves, and a total of 488 differentially expressed genes were identified, and one drought-related genetic locus was discovered, which provided a new strategy for breeding drought-tolerant maize [38]. In our study, a total of 60.35 Gb of clean data were obtained by transcriptome sequencing, and the proportion of valid reads in each sample was concentrated at around 96.97%. By screening the classified DEGs with GO, KEGG and fine functional annotation, most DEGs were significantly involved in transcriptional regulation, with dna-triggered positive and negative regulation of transcription, redox process, and protein phosphorylation. A transcriptome study related to cold stress was previously performed on other crops, and the GO term ‘response to stimulus’ was emphasized by GO analysis, from which some cold stress genes were identified [39,40]. GO analysis in this study showed that the defense process of drought stress include: “GO:0009737: response to abscisic acid”, “GO:0006952: defense response” and “GO:0050832: defense response to fungi” by KEGG enrichment analysis in “ko00500: starch and sucrose metabolism”, “ko01200: carbon metabolism”, “ko04626: interaction between plant and pathogen” and “ko04075: plant hormone signaling” (Figure 3 and Figure 4). Soybean (*Glycine max* L.) treated with drought and salt stress RNA-seq sequencing showed that 26 GmPP2A-B genes were significantly up-regulated in soybean under salt stress and drought stress, respectively. Since protein phosphatase 2A plays an important role in the regulation of cellular ROS signaling, the results suggest that the GmPP2A-B gene can promote drought resistance in soybean by regulating the ROS signaling pathway [34]. In this study, according to transcriptomic analysis, we found that the GO items “GO:0016209: antioxidant activity” and “GO:0055114: redox process” could maintain the redox balance, thus reducing cell damage from oxidative stress and regulating the occurrence of drought stress [41].

miRNAs are a class of endogenous non-coding RNAs with regulatory functions found in eukaryotes, with a size of about 18~25 nucleotides. Gene expression is often silenced by inhibiting translation and accelerating the degradation of target mRNA [2]. miRNA interacts with the signaling pathway of growth regulators, which is not only a regulator of plant growth and development but also participates in regulating the phenotypic plasticity induced by various environmental stimuli such as light, temperature, nutrients, etc., and promotes plant evolution and adaptation. Due to sedentary growth, plants are often exposed to adverse conditions such as drought, high salinity, extreme temperatures, and deficiencies in heavy metals and nutrients, which are major factors limiting the geographical distribution of plants and crop yields. Among them, miRNA-mediated gene expression regulation is an important mechanism for plants to cope with abiotic stresses [3,4]. miRNAs play an important role in the response of plants to drought stress [4,5,6]. Under drought stress, plants can achieve this by changing their physiological state and regulating related genes. Therefore, it is of great significance to study the molecular mechanism of plant response to drought stress by studying miRNAs [7,8]. Micro RNAs (miRNAs) are involved in diverse biological processes including adaptive response towards abiotic stresses. To unravel small RNAs and more specifically miRNAs that can potentially regulate determinants of abiotic stress tolerance, next generation sequencing of *B. juncea* seedlings subjected to high temperature, high salt and drought conditions was carried out. Using the generated sequence and other publically available Brassica genomic/transcriptomic resources as mapping reference, 126 novel (not reported in any plant species) were discovered for the first time in *B. juncea*. The expression of selected conserved and novel miRNAs under conditions of different abiotic stresses was revalidated through universal TaqMan based real time PCR. Putative targets of identified conserved and novel miRNAs were predicted in *B. rapa* to gain insights into functional roles manifested by *B. juncea* miRNAs. Investigation of gene ontologies linked with targets of known and novel miRNAs forecasted their involvement in various biological functions [42]. In this study, we used miRNA-seq analysis to identify differentially regulated genes under drought stress conditions, and we used DEseq software to compare gene expression between groups of *Brassica rapa* under three growing conditions. 141, 180, and 102 differentially expressed miRNAs (DEMIs) were identified between D and CK, R and CK, and R and D comparisons, respectively. Through the analysis of the main pathways of differentially expressed miRNA, the results showed that most miRNA target genes were mainly enriched in “ko04626: plant-pathogen interaction”, “ko00190: oxidative phosphorylation”, “ko00860: porphyrin and chlorophyll metabolism”, and “ko04141: protein processing in the endoplasmic reticulum” (Figure 8B, Supplementary Table S8).

Kang et al. [28]. identified the pathways of “phenylpropanoid biosynthesis”, “plant hormone signaling”, and “starch and sucrose metabolism” necessary for potatoes to cope with alkaline stress through miRNA-mRNA integration analysis. It was found that miR4243-x and novel-m064-5p were involved in the response of potato to alkaline stress through the negative regulation of shikimic acid O-hydroxy cinnamicyl transferase (HCT) and sucrose phosphate synthetase (SPS) genes, respectively [42]. A correlation analysis of key DEGs and DEGs showed that many DEGs play a direct or indirect regulatory role in the metabolism of DEGs. The comprehensive analysis of DEGs showed that the starch and sucrose pathways were the key pathways for the ABA reaction. To explore the relationship between miRNA and mRNA under drought stress. In this study, microRNA sequencing data and mRNA transcriptome data were jointly analyzed, and DEMIs were selected by DEGs-targeted binding in each group of treatments to form a negative regulatory targeting relationship. Further analysis of association regulation showed that BraA06g033670 was involved in the regulation and expression in both BR18R_1_D vs. BR18R_1_CK and BR18R_1_R vs. BR18R_1_D. At the same time, BraA06g035670 was expressed in BR18R_1_D vs. BR18R_1_CK and BR18R_1_R vs. BR18R_1_CK. The classified DEGs were screened together with GO, KEGG and fine functional annotation and analyzed by miRNA in their target mRNA association regulation. The analysis showed that most of the target mRNA was mainly enriched in ko00710: carbon fixation of the Calvin cycle, ko00500: starch and sucrose metabolism, ko04075: phytohormone signal transduction GO entries were enriched in GO:0019253: reduced pentose phosphate cycle, GO:0009409: Response to cold, GO:0042742: Gene defense response to bacteria in biological processes; In the cellular components, target mRNA is enriched in the chloroplast, chloroplast, cell–matrix, cell cytoplasm and cell nucleus. The enriched molecular functions were mainly related to ATP binding, riboto-diphosphate carboxylase activity, monooxygenase activity, kinase activity, and protein binding. The expression heatmap analysis showed that BraA05g023780 and BraA05g040570, BraA09g063780, BraA01g030200 and BraA06g033670 were up-regulated about 11, 46.8, 9.7, 28.7, 2.7 times in BR 18 R_1_R vs. BR18R_1_D, respectively. This means that the expression of drought-affected genes returned to a “normal” state after rewatering treatment. In conclusion, it is clarified that multi-omics techniques play an indispensable role in revealing abiotic stresses such as the regulation of plant growth, development and stress tolerance.

## 4. Materials and Methods

### 4.1. Plant Material, Drought Stress Treatment, and Sample Collection

The drought-resistant variety Longyou 7 (formerly BR18R-1) screened by the *B. rapa* research group of Gansu Agricultural University (Lanzhou, Gansu, China) was used as the test material. Healthy and viable seeds were carefully chosen for disinfection. Seeds were then rinsed with distilled water and placed on moist filter paper to facilitate germination. After 5 days, the germinated seeds were transplanted into nutrient soil and cultivated at a temperature of 25 °C. The soil moisture level was maintained at 80%. The plants were allowed to grow until they reached the 3–4 leaf stage. At this point, a drought treatment was initiated. No treatment as control group (CK), while the treatment group was subjected to simulated drought stress using a 17% PEG solution for 7 days (D). The effective soil moisture level was maintained at 10% during this period. Subsequently, the drought-stressed seedlings were rewatered for 4 days (R) to restore the health of leaves [43]. The samples of rapeseed leaves were collected after various treatments. For each treatment, three biological replicates were taken and preserved at −80 °C.

### 4.2. mRNA Library Construction and Sequencing

RNA extraction was performed from rape leaves using Trizol reagent (Invitrogen, Carlsbad, CA, USA) according to the manufacturer’s protocol. The Bioanalyzer 2100 and RNA 6000 Nano LabChip Kit (Agilent, Santa Clara, CA, USA) were used to analyze the RNA integrity purity. The RNA samples were required to have a RIN value greater than 7.0. Around 10 μg of total RNA, which represents a particular type of Leaf tissue, was extracted from Poly(A) mRNA using magnetic beads connected with poly-T oligonucleotide (Invitrogen). After purification, the mRNA is fragmented into small pieces using divalent cations at high temperatures. The cleaved RNA fragments were then reverse transcribed according to the protocol of the mRNA Seq sample Prep Kit (Illumina, San Diego, CA, USA) to create a final cDNA library with an average insert size of 300 bp (±50 bp) for paired-end libraries. We then performed paired-end sequencing on an Illumina Novaseq 6000™ (LC Sciences, Houston, TX, USA) following the vendor’s recommended protocol [44,45]

### 4.3. Transcriptome Sequencing Process and Quality Control of Sequencing Data

After the total RNA quality check was passed, eukaryotic mRNA was enriched using magnetic beads attached to Oligo(dT). The extracted mRNA was randomly interrupted into short fragments by fragmentation buffer, and the fragmented mRNA was used as a template to synthesize a strand of cDNA with six-base random primers (Random hexamers), and then buffer, dNTPs, RNaseH and DNA Polymerase I were added for two-stranded cDNA synthesis. AMPure XP beads purified the double-stranded product, using T4 DNA polymerase and Klenow DNA polymerase activity to repair the sticky end of the DNA to blunt end, adding base A to the 3′ end and adding adapters, AMPureXP beads for segment selection, and finally PCR amplification to obtain the final sequencing library. After the library quality inspection was qualified, Illumina Novaseq™ 6000 (Illumina, Inc., San Diego, CA, USA) was used for sequencing, and the sequencing read length was 2 × 150 bp (PE150) [46].

### 4.4. Assembling, Sequencing, and Data Preprocessing

The reference genome for *B. rapa* was obtained through sequencing at Gansu Agricultural University (NCBI accession number: PRJNA1150086), which exhibited a significant degree of homology. In order to ensure accurate and trustworthy analysis results, the raw data after sequencing including the removal of sequencing connectors and low-quality sequencing data, and the normalization of FPKM, etc., are required. The original sequencing data underwent quality control using Fast QC software v0.12.1, resulting in the acquisition of clean reads [46]. These clean reads were then compared to the reference genome using hisat software 2.2.1. Transcript splicing and merging were performed using StringTie software v2.2.3, the FPKM value of gene expression was calculated using cufflinks software version 1.47, the number of reads on the aligned gene in each sample was obtained using Htseq software version 2.0.5 (Heidelberg, Germany), and the FPKM value of gene expression was calculated using cufflinks software [23,28].

### 4.5. miRNA Sequencing

The total RNA of the samples was isolated and purified as above, and the concentration and purity of the total RNA were checked using the NanoDrop ND-1000 (NanoDrop, Wilmington, DE, USA), and the fragment integrity was measured using the Bioanalyzer 2100 (Agilent, CA, USA). Total RNA and 3′ adapters were denatured at 70 °C for 2 min, ligated with T4 RNA ligase 2 (NEB, M0351L, Ipswich, MA, USA), and purified overnight at 16 °C. RTP reagent was added and incubated at 37 °C for 30 min to neutralize and remove the single-stranded DNA 3′ adapter from the reaction. The ligation product was ligated with T4 RNA ligase 1 (NEB, Cat. No. M0204L, USA), and the RNA 5′ adapter was connected and purified after 1 h incubation at 37 °C. SuperScript™ II Reverse Transcriptase (Thermo, 18064014, Waltham, MA, USA) was utilized for the reverse transcription reaction, which was incubated at 50 °C for 1 h and then at 80 °C for 10 min. Phusion^®^ Ultra-Fidelity DNA Polymerase (NEB, Cat. No. M0530L, USA) was employed for the synthesis of two strands, and the resulting product was amplified and introduced with a specific Index. This process involved pre-denaturing at 98 °C for 30 s, denaturing at 98 °C for 10 s, annealing at 60 °C for 30 s, and extending at 72 °C for 15 s, for a total of 10 to 16 cycles. The mixture was then held at 72 °C for 5 min, and the miRNA library was purified and enriched through PAGE electrophoresis. SE50 single-ended sequencing was performed using the Illumina Hiseq 2500 as per standard operation (NCBI accession number: PRJNA1150664) [44,47].

### 4.6. Data Processing

DESeq software was used to analyze the differential expression of the treatment and the control group, and the differential genes were screened by the negative binomial distribution test (NB) [48]. StringTie assembled and quantitatively completed genes were analyzed for differences using edgeR version 3.10 (threshold for significant differences was log2foldchange ≥ 1, *p* < 0.05). Genes with *p*-value less than 0.05 and fold-change greater than 2 were screened as differentially expressed genes. Gene ontology (GO) and KEGG (Kyoto Encyclopedia of Genes and Genomes) enrichment analysis was performed to describe the functions and pathways mainly involved in the differentially expressed genes [49,50]. Based on the transcriptome level, SNP sites in coding regions were analyzed. Based on the results of Hisat comparison of each sample with the reference genome using samtools software 1.12 for mpileup processing, the possible SNP and INDEL information of each sample was then annotated using annovar 24.10.2019 [51]. Qualitative analysis of variable shear events was performed separately for each sample using ASprofile software version b-1.0.4 on the gene model predicted by Stringtie (transcript.gtf).

## 5. Conclusions

In this study, 14–15 differentially expressed mRNAs (DEGs) were identified across three groups through transcriptome and miRNA sequencing of the drought-resistant variety Longyou 7. GO annotation and KEGG analysis revealed that these DEGs are primarily involved in growth metabolism, stress responses, transcriptional regulation and various biosynthetic pathways. Several miRNAs, such as gma-miR6300 and gra-MIR8731-p3_2ss13GA18GA, were found to potentially regulate drought resistance in rapeseed by negatively influencing the expression of multiple mRNAs. Key pathways affected include starch and sucrose metabolism, amino acid biosynthesis and carbon metabolism. These findings provide valuable information on the roles of genes and miRNAs in rapeseed response to abiotic stress, particularly drought, and lay a foundation for future research on improving drought resistance in *B. rapa*.

## Data Availability

Data are contained within the article and Supplementary Materials.

## Supplementary Materials

The supporting information can be downloaded at: https://www.mdpi.com/article/10.3390/ijms251810098/s1.
