# Supplementary material for: Integrated miRNA and mRNA Transcriptome Analysis Reveals Regulatory Mechanisms in the Response of Winter Brassica rapa to Drought Stress
Source: Int J Mol Sci. 2024 Sep 20;25(18):10098. doi: 10.3390/ijms251810098 (PMC11432419; doi:10.3390/ijms251810098)
Supplement: Supplementary file 1 [file ijms-25-10098-s001.zip › ijms-3199473-supplementary.pdf]

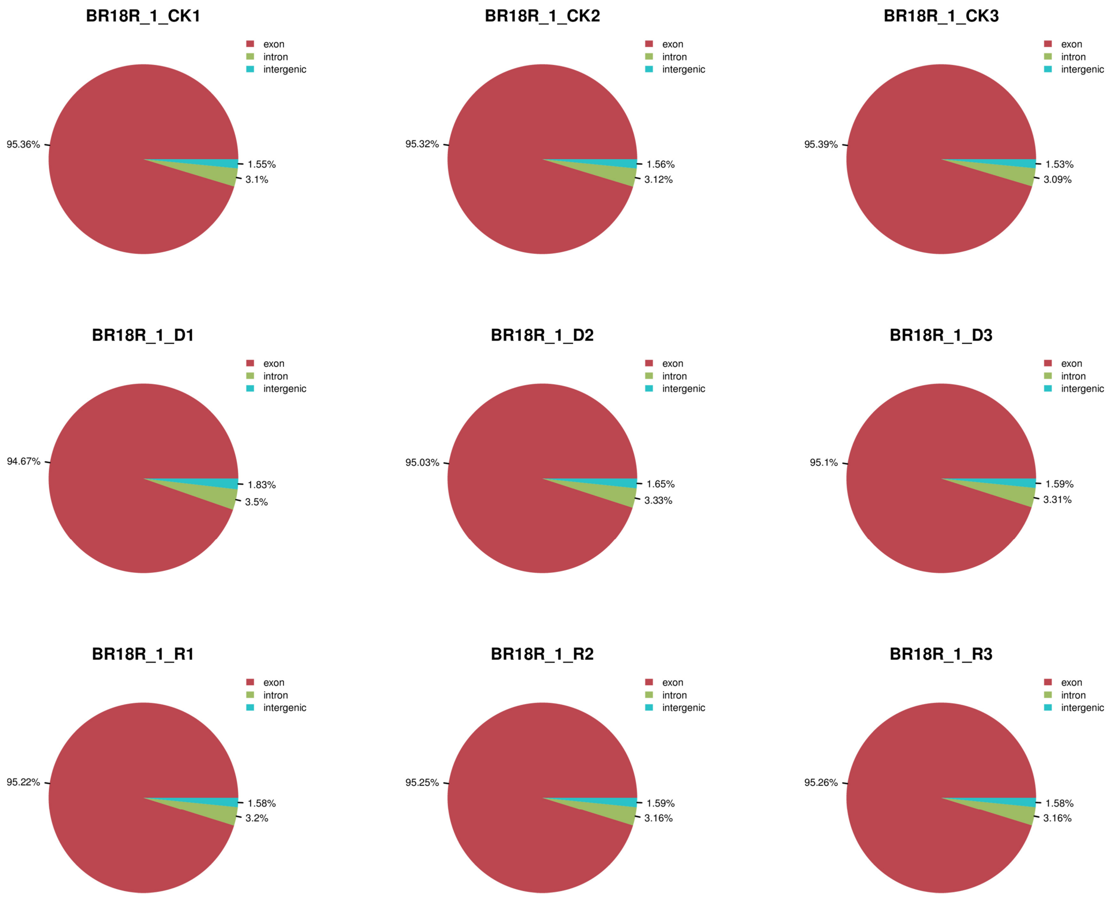

Figure S1. Distribution of reference genome comparison regions. 1, 2 and 3 are untreated (CK), 4, 5 and 6 are drought stress treatments (D), and 7, 8 and 9 are rehydration treatments (R). The genome was categorized into exonic, intergenic, and intronic regions in the figure, and the region size was according to the percentage of Reads compared to the corresponding region in all Mapped Reads.

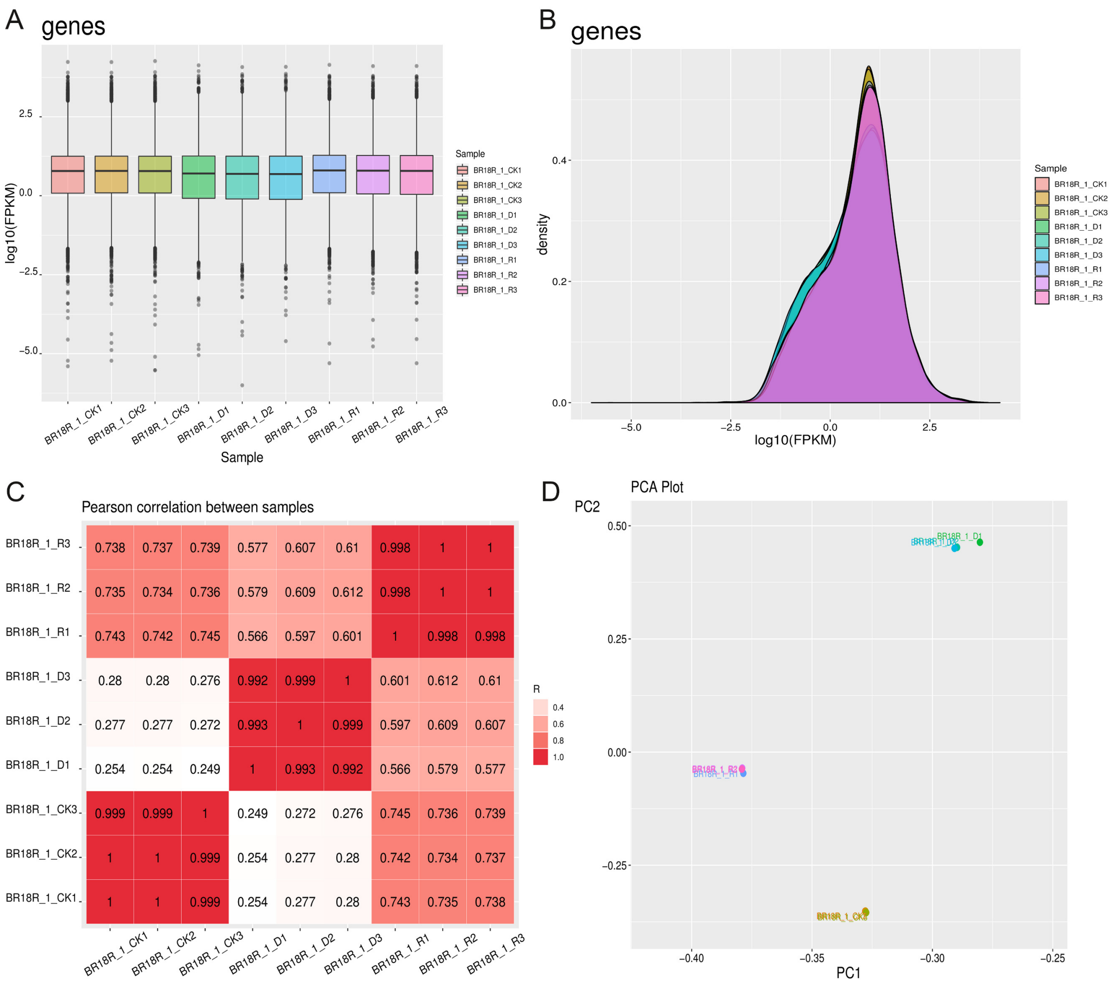

Figure S2. Distribution statistics of gene expression values, correlation heatmap and gene principal component analysis for each sample. A is the FPKM container line diagram. The different colors in the figure represent different ranges of FPKM values, with the horizontal axis representing samples and the vertical axis representing the number of protein coding genes. B is the FPKM density distribution curve. The curves of different colors in the figure represent different samples. The horizontal axis of the points on the curve represents the logarithmic value of the corresponding sample FPKM, and the vertical axis of the points represents the probability density. C, The values on each color block on the heatmap represent the correlation between the two samples along the horizontal and vertical axes corresponding to the color block. The larger the values, the higher the correlation. D, Different coordinates represent different principal components, percentages represent the contribution of corresponding principal components to sample differences, each point represents a sample, and samples from different groups are represented by different colors and shapes.

Supplementary Table S1 Sequencing data statistics

Sample	Raw Data	Base	Valid Data	Base	Valid Ratio(reads)	Q20 %	Q30 %	GC content%
BR18R_1_CK1	43309258	6.50 G	42193052	6.33 G	97.42	99.96	97.82	47
BR18R_1_CK2	40539710	6.08 G	39528986	5.93 G	97.51	99.96	97.78	47
BR18R_1_CK3	41684026	6.25 G	40319796	6.05 G	96.73	99.96	97.79	47
BR18R_1_D1	38943996	5.84 G	37870946	5.68 G	97.24	99.96	97.77	46
BR18R_1_D2	46341852	6.95 G	45016094	6.75 G	97.14	99.97	98.06	46.50
BR18R_1_D3	48433986	7.27 G	46770290	7.02 G	96.57	99.97	98.04	46.50
BR18R_1_R1	44155214	6.62 G	42105086	6.32 G	95.36	99.97	97.97	47
BR18R_1_R2	46265824	6.94 G	45092684	6.76 G	97.46	99.97	97.99	47
BR18R_1_R3	52671024	7.90 G	51256528	7.69 G	97.31	99.97	97.99	47

Sample: Sample Name, Raw Data Reads: Number of reads in raw data, Valid Data Reads: Number of reads in valid data, Valid Ratio%: Proportion of valid reads. Base: Data Size, Q20%: Proportion of bases with quality values ≥ 20 (sequencing error rate < 0.01), Q30%: Proportion of bases with quality values ≥ 30 (sequencing error rate < 0.001), GC content%: Proportion of GC content.

Supplementary Table S2 Reference genome alignment of reads statistics

Sample	Valid reads	Mapped reads	Unique Mapped reads	Multi Mapped reads	Reads map to sense strand	Reads map to antisense strand
BR18R_1_CK1	42193052	34594977(81.99%)	28319812(67.12%)	6275165(14.87%)	17092557(40.51%)	16854419(39.95%)
BR18R_1_CK2	39528986	32384851(81.93%)	26491114(67.02%)	5893737(14.91%)	16003783(40.49%)	15775548(39.91%)
BR18R_1_CK3	40319796	33035449(81.93%)	27098239(67.21%)	5937210(14.73%)	16319940(40.48%)	16098957(39.93%)
BR18R_1_D1	37870946	31027754(81.93%)	25265950(66.72%)	5761804(15.21%)	15351807(40.54%)	15122162(39.93%)
BR18R_1_D2	45016094	37187556(82.61%)	30463638(67.67%)	6723918(14.94%)	18374854(40.82%)	18146574(40.31%)
BR18R_1_D3	46770290	38621304(82.58%)	31679273(67.73%)	6942031(14.84%)	19073257(40.78%)	18856324(40.32%)
BR18R_1_R1	42105086	34588471(82.15%)	28695384(68.15%)	5893087(14.00%)	17036199(40.46%)	16861626(40.05%)
BR18R_1_R2	45092684	37174060(82.44%)	30626949(67.92%)	6547111(14.52%)	18324644(40.64%)	18104889(40.15%)
BR18R_1_R3	51256528	42268809(82.47%)	34881865(68.05%)	7386944(14.41%)	20830747(40.64%)	20590058(40.17%)

Sample: sequencing library name, Valid reads: quality-controlled data, Mapped reads: the number of reads that can be aligned to the genome, Unique Mapped reads: the number of reads that can only be uniquely aligned to one position of the genome, Multi mapped reads: the number of reads that can be aligned to multiple positions of the genome, Reads map to sense strand: the statistical number of reads aligned to the sense strand on the genome, Reads map to antisense strand: the statistical number of reads aligned to the antisense strand on the genome.

Supplementary Table S9. Combined analysis of BR18R_1_D VS BR18R_1_CK DEMIs and DEMs targeting

Gene ID	miRNA ID
BraA04g010950	gma-miR6300
BraA06g033670	PC-3p-13322_404
BraA04g014860	gma-miR6300
	gma-miR6300_R+1
BraA01g042490	bra-MIR9552a-p3
BraA06g035670	gra-MIR8731-p3_2ss13GA18GA
BraA03g043710	mtr-miR171b_L-1
BraA02g004600	bra-miR9558-3p
	gra-MIR8731-p3_2ss13GA18GA
BraA03g035490	gra-MIR8731-p3_2ss13GA18GA

Combined analysis of BR18R_1_R VS BR18R_1_CK DEMIs and DEMs targeting

Gene ID	miRNA ID
BraA10g029260	bna-MIR397b-p3
BraA05g024150	bna-MIR166c-p5
BraA04g002530	bra-miR5720
BraA09g060240	ath-miR159c_R-1_1ss19CT
BraA02g041250	ath-MIR414-p5_1ss18CT
	bra-miR156a-3p
	bra-miR156b-3p_1ss15TC
	bra-miR172c-3p_R-1
	bra-miR172d-3p
	gma-miR6300
	gma-miR6300_R+1
	gma-miR6300_R+2
BraA07g008810	PC-5p-740_6264
BraA06g035670	gra-MIR8731-p3_2ss13GA18GA

Combined analysis of BR18R_1_R VS BR18R_1_CK DEMIs and DEMs targeting

Gene ID	miRNA ID
BraA05g023780	gra-MIR8731-p3_2ss13GA18GA
BraA05g040570	gra-MIR8731-p3_2ss13GA18GA
	gra-MIR8731-p3_2ss13GA18GA
BraA09g063780	ghr-MIR482b-p3_2ss14GC18AG
	ghr-MIR482b-p5_2ss14GC18AG
BraA01g030200	hbr-MIR6173-p3_1ss4CT
	cas-MIR408-p5
BraA06g033670	PC-3p-13322_404
